# Myocardial perfusion imaging by single-photon emission tomography (MPI SPECT) versus Instantaneous wave-free ratio (IFR) for assessment of functional significance of intermediate coronary artery lesions

**DOI:** 10.1186/s43044-019-0031-1

**Published:** 2019-12-29

**Authors:** Osama Ahmed Amin, Yasser Ahmed Abdel Hady, Mohammad Ahmad Nour El-Din Esmail

**Affiliations:** Cardiology Department, Beni-Suef University, Beni Suef, Egypt

**Keywords:** Myocardial perfusion imaging, Instantaneous wave-free ratio, Fractional flow reserve

## Abstract

**Background:**

The aim of our work was to compare the myocardial perfusion imaging by single-photon emission tomography (MPI-SPECT) as a non-invasive, relatively non-expensive test versus the instantaneous wave-free ratio (IFR) for the evaluation of functional significance of the borderline coronary artery lesions in the view of results of fractional flow reserve (FFR) which is considered the gold standard reference test.

**Results:**

Our study was conducted in the Cardiology Department. It included 50 patients with borderline coronary artery lesions; they underwent physiological evaluation by stress/rest myocardial perfusion imaging using followed by an invasive physiological assessment by Instantaneous wave-free ratio (IFR) and Fractional flow reserve (FFR). Finally, the results of both SPECT MPI and IFR were compared to FFR as a gold standard reference. There was a strong (kappa = 0.754) significant (*P* value < 0.001) agreement between the MPI results and FFR results and the overall agreement was 88%. The sensitivity of the MPI was 81.8%, the specificity was 92.9%, the positive predictive value was 90%, the negative predictive value was 86.7%, the positive likelihood ratio was 11.45, and the negative likelihood ratio was 0.20. There was a strong (kappa = 0.918) significant (*P* value < 0.001) agreement between the IFR results and FFR results and the overall agreement was 96%. The sensitivity of the IFR was 90.9%, the specificity was 100%, the positive predictive value was 100 %, the negative predictive value was 93.3%, and the negative likelihood ratio was 0.09.

**Conclusions:**

The instantaneous wave-free ratio (IFR) may be a valid alternative to fractional flow reserve to assess the functional significance of intermediate coronary lesions. The myocardial perfusion imaging may be an alternative, non-invasive, relatively non-expensive test for the evaluation of the physiological significance of intermediate coronary lesions.

## Background

Invasive angiography is the reference test for the diagnosis of CAD. However, the relationship between the angiographic stenosis severity and the coronary blood flow is complex. The visual assessment of stenosis severity is subjective and correlates poorly with physiological significance [1]. Unfortunately, coronary angiography has many pitfalls and limitations that may impair the judgment of stenosis severity and then affect decision-making regarding intervention. The limitations of coronary angiography include the interpretation is highly subjective (inter- and intra-observer variability) [2–4], comparing to normal reference segment can be fallacious in diffuse disease, the eccentric lesions have a varying appearance of severity in different views [5], and several artifacts can give a false impression about severity. In addition to all of the previous limitations that make conventional coronary angiography correlates poorly with physiological significance, assessment of intermediate or borderline coronary lesions is another common challenge in routine practice. The physiological assessment of coronary lesions has become a cornerstone practice in the current clinical cardiology guidelines. Myocardial perfusion imaging by single-photon emission tomography (MPI–SPECT) is used for a long time to detect reversible ischemia, quantify defect sizes and help clinical decisions of interventions. It has been even used for early validation of invasive physiological assessment by fractional flow reserve (FFR). Despite this fact, the patient outcome studies performed on the role of invasive physiological assessment by FFR created a prestigious position and enlightened a hot spot on FFR use for functional assessment of coronary lesions, and so FFR is now considered the gold standard test for this purpose. However, fractional flow reserve (FFR) is considered a relatively expensive test, especially with limited resources in our country, and also implies the use of pharmacological hyperemic agents (e.g., adenosine) which are relatively not easily available in our country. The development of Instantaneous wave-free Ratio (IFR) as a relatively new invasive method for physiological assessment of coronary lesions without the use of pharmacologic hyperemic agents has solved the problem partially but is still considered a relatively expensive test with our limited resources. On the other hand, myocardial perfusion imaging by single-photon emission tomography (MPI-SPECT) is a non-invasive, relatively non-expensive test and is well supported in the national health insurance systems unlike the FFR and IFR which are much less supported by the insurance programs and so their use is usually limited even when needed for decision-making.

## Aim of the work

The aim of this work was to compare the myocardial perfusion imaging by single-photon emission tomography (MPI-SPECT) as a non-invasive, relatively non-expensive test versus the instantaneous wave-free ratio (IFR) for the assessment of functional significance of the intermediate coronary artery lesions in the view of results of fractional flow reserve (FFR) which is considered the gold standard reference test.

## Methods

This study prospectively recruited 50 ischemic heart disease patients presented to the cardiology department with intermediate (40–70%) lesions in coronary arteries to compare the results of single-photon emission tomography myocardial perfusion imaging (MPI-SPECT) versus instantaneous wave-free ratio (IFR) in the view of results of fractional flow reserve (FFR) as a gold standard reference.

The protocol was approved by the committee of the medical ethics of the cardiology department in March 2016 and informed written consent was obtained from all the patients. The pre-procedural assessment was done in the cardiology outpatient clinic.

Inclusion criteria:

Patients with angiographically borderline coronary artery stenosis (40–70%) in a single coronary artery were assessed by at least two expert operators. The following patients were excluded from our study:

I. Patients with certain limitations to FFR:

A. Acute coronary syndrome:

1. STEMI.
2. Non-STE ACS.

B. Patients with marked left ventricular hypertrophy: as microvascular abnormalities may be present and impair the results of FFR [1].
C. Patients with tandem lesions: FFR pullback has limited value for assessment of tandem successive coronary lesions [6].

II. Contraindications to stress—Tc-99 MPI: [7]

1. Iodine 131 treatment within the last 3 months.
2. Indium 111 or gallium 67 scan within the last month.
3. Tc-99 imaging within 2 days.
4. Pregnant ladies.
5. Congestive heart failure.
6. Severe hypertension (blood pressure > 200/110 mmHg).
7. Uncontrolled cardiac arrhythmias (causing symptoms or hemodynamic compromise).
8. Severe valvular lesions.
9. Acute pulmonary embolism.
10. Other acute cardiac conditions (myocarditis, pericarditis, or aortic dissection.
11. Significant pulmonary hypertension.

III. Conditions that may cause a controversial interpretation of SPECT MPI:

Previous myocardial infarction and Multi-vessel disease.

IV. Clinically unstable patients for any reason (e.g., severe infections).

The patients were subjected to the following:

Informed consent: According to the World Medical Association (WMA) Declaration of Helsinki 2013 [8]. For each patient, history was taken and physical examination was performed. The electrocardiographic recording was done to each patient to detect any baseline abnormalities. All the patients were subjected to detailed conventional M-mode and 2D transthoracic echocardiographic examination and Doppler study using standard parasternal and apical views following the recommendations of the European Association of Cardiovascular Imaging [9] using an imaging system equipped with a 2–4 MHz transducer, to assess left ventricular dimensions, posterior wall (PWT), septal wall thickness(SWT), fractional shortening (FS), and ejection fraction (EF). All the patients underwent stress/rest Tc99m SestaMIBI SPECT 2-day protocol to study myocardial perfusion [7] using Siemens Symbia-E dual head system- Syngo MI workplace 2011-Emory Cardiac toolbox software system.

- Myocardial perfusion image (MPI) protocol: [7, 10]
- Two-day protocol, stress, and rest Tc99m sestaMIBI myocardial perfusion SPECT was performed.
- Patients were exercised on a treadmill according to the standard Bruce protocol [7].
- Patients with contraindications for exercise test underwent pharmacologic stress test (Dobutamine stress test).
- Technetium-99m Sestamibi 20 mCi was injected at peak exercise. Then, the patient was instructed to eat a fatty meal to decrease hepatic activity during imaging.
- After 30–60 min, imaging acquisition started. Imaging was in the supine position.
- On the second day, patients were re-injected by 20 mCi Tc99m sestaMIBI and re-imaged after 30–60 min with the same protocol. The image processing and reconstruction were performed according to the American Society of Nuclear Cardiology guidelines [7, 10].
- Image analysis was performed using a previously validated automated program that determines the extent and severity of LV perfusion defect size and the extent of reversible (ischemia) or fixed (scar) resting hypoperfusion and LVEF.
- SPECT MPI results were considered positive when the defect size ≥ 10% in the territory of interest.

I. Instantaneous wave-free ratio (IFR) and Fractional Flow Reserve (FFR): [1, 6, 11]

- Each patient underwent IFR and FFR measurements, the following technique was applied:
  - Philips Volcano system was used for IFR & FFR measurements using prime-prestige pressure wires.
  - Routine preparation:
  - Patients were instructed to stop caffeine intake 24 h before the procedure as caffeine is considered as adenosine receptor antagonist that may interfere with adenosine-induced hyperemia and affects FFR results [12]. No anesthesia was required.
  - Access: routine trans-femoral or trans-radial approach for cardiac catheterization was applied.
  - Diagnostic coronary angiography was done with localization of the site of stenosis of interest.
  - Calibration of pressure systems: the pressure lines were flushed with saline. Then, the “zero- reference” was recorded.

The sensor was then positioned 2 mm distal to the tip of the guiding catheter and we flushed the guiding catheter using saline. At that site, both pressures were identical. If the two pressures were not equal, the measured pressures had to be equalized electronically, using that function of the console. If there was ostial stenosis, this should be performed with the pressure sensor positioned in the aorta. The sensor was then manipulated in the distal part of the artery. In any case, the sensor was placed 3 cm distal to the stenosis to be assessed, a distance where the post-stenotic laminar flow is restored. The pressure transducer was located approximately 3 cm proximal to the distal tip of the wire and it could be seen by fluoroscopy.

- Instantaneous wave-free ratio (IFR) was calculated in a computerized manner independent of any induced hyperemia. An IFR value lower than 0.89 was considered positive indicating hemodynamically significant stenosis and IFR value higher than 0.89 was considered negative indicating stenosis that is not hemodynamically significant [1, 12].
- To measure fractional flow reserve (FFR); once the transducer was distal to the stenosis, a hyperemic stimulus was administered by injection through the guide catheter, and the FFR is monitored for a significant change.
- Intracoronary adenosine was used: 100 μg bolus in the right coronary artery or 200 μg bolus in the left coronary artery [13]. Intracoronary adenosine was a well-accepted alternative to intravenous adenosine to achieve maximal hyperemia for FFR measurements [121]. Using intracoronary adenosine was used to overcome the higher costs and the relative non-availability of adenosine in Egypt.
- The mean arterial pressures from the pressure wire transducer and from the guide catheter were then used to calculate FFR [1, 13].
- In the case of adenosine-induced hyperemia: an FFR value lower than 0.8 was considered positive indicating significant stenosis and FFR value higher than 0.8 was considered negative indicating stenosis that was not hemodynamically significant [1, 6].

## Statistical analysis

Analysis of the data was performed using SPSS v. 23 (Statistical Package for Social Science) for Windows. The description of the variables was presented as follows:

- The description of quantitative variables was in the form of mean, standard deviation (SD), median, and range (min-max).
- The description of qualitative variables was in the form of numbers (no.) and percent (%).
- The data were explored for normality using Shapiro/Kolomogrov tests of normality.
- Kappa agreement was done to test the agreement between the MPI and the IFR with the gold standard FFR.
- Pearson correlation was conducted to test the correlation between the MPI and the IFR with the gold standard FFR
- Kappa and correlation coefficient *r* was considered mild till 0.3, moderate till 0.6 and strong for more than 0.6.
- ROC curve was used to assess the cut-off points of MPI and IFR at which we can find the best prediction of positive cases by FFR.

The results were assessed in the form of *P* value that was classified into:

- Non-significant when *P* value > 0.05
- Significant when *P* value ≤ 0.05
- Highly significant when *P* value ≤ 0.001

## Results

The study was conducted in the Cardiology Department, in the period between January 2017 and June 2019. It included 50 patients with stable coronary artery disease and intermediate coronary artery lesions, they underwent physiological evaluation by stress/rest myocardial perfusion imaging using single-photon emission tomography (SPECT-MPI) followed by an invasive physiological assessment by instantaneous wave-free ratio (IFR) and fractional flow reserve (FFR). Finally, the results of both SPECT MPI and IFR were compared to FFR as a gold standard reference Table 1 shows Age and sex distribution of the studied patients, Table 2 shows Distribution of co-morbidities of medical importance among the studied patients, Table 3 shows Family history and special habits of medical importance among the studied patients, Table 4 shows Description of body morphology of medical importance among the studied patients, Table 5 shows Coronary lesions severity of the affected vessels in the studied patients, Table 6 shows Distribution of the target vessel among the studied patients, Table 7 shows Description of the spectroscopy MPI defect size of the affected vessels in the studied patients, Table 8 shows Description of the spectroscopy IFR of the affected vessels in the studied patients and Table 9 shows Description of the spectroscopy FFR of the affected vessels in the studied patients.

**Table 1:** Age and sex distribution of the studied patients

Parameters	Values
Age	
Mean ±SD	55.28 ±8.6
Range(min-max)	(34-68)
Median	56
Sex no. (%):	
Males	36(72%)
Females	14(28%)

Scale data were presented as mean ± SD and categorical data were presented as number (%)

**Table 2:** Distribution of co-morbidities of medical importance among the studied patients

Co-morbidities	Number *N* = 50	Percent 100%
HTN		
Positive	32	64.0
Negative	18	36.0
DM		
Positive	26	52.0
Negative	24	48.0
Dyslipidemia		
Positive	40	80.0
Negative	10	20.0

Data were presented as number and percen

**Table 3:** Family history and special habits of medical importance among the studied patients

co-morbidities	Number N=25	Percent 100%
Family history		
Positive	32	64.0
Negative	18	36.0
Smoking		
Smokers	26	52.0
Non-smokers	24	48.0

Data were presented as number and percent

**Table 4:** Description of body morphology of medical importance among the studied patients

Parameters	Values
Weight	
Mean ± SD	86.6 ± 17.9
Range(min-max)	(55–130)
Median	85
Height	
Mean ± SD	1.69 ± 0.63
Range(min-max)	(1.6–1.85)
Median	1.7
BMI	
Mean ±SD	30.1 ± 6.2
Range(min-max)	(20.2–46.6)
Median	29.4

Scale data were presented as mean ± SD

**Table 5:** Coronary lesions severity of the affected vessels in the studied patients

Size	Values
Mean ± SD	58.4 ± 8
Range(min-max)	(50–70)
Median	60

Scale data were presented as mean ± SD

**Table 6:** Distribution of the target vessel among the studied patients

The target vessel	Number (%)
LAD	32 (64%)
LCX	10 (20%)
RCA	8 (16%)

**Table 7:** Description of the spectroscopy MPI defect size of the affected vessels in the studied patients

Parameters	Values
Defect size of only positive MPI:	
Mean ± SD	15.6 ± 4.5
Range (min-max)	(10–22)
Median	14
MPI results no. (%):	
Positive	20(40)
Negative	30(60)

Scale data were presented as mean ± SD and categorical data were presented as number (%)

**Table 8:** Description of the spectroscopy IFR of the affected vessels in the studied patients

Parameters	Values
IFR value	
Mean ± SD	0.89 ± 0.094
Range (min-max)	(0.65–1.05)
Median	0.92
IFR results no. (%):	
Positive	20(40)
Negative	30(60)

Scale data were presented as mean ±SD & categorical data were presented as number (%)

**Table 9:** Description of the spectroscopy FFR of the affected vessels in the studied patients

Parameters	Values
FFR value	
Mean ± SD	0.796 ± 0.1007
Range(min-max)	(0.60–0.96)
Median	0.83
FFR results no. (%):	
Positive	22(44)
Negative	28(56)

Scale data were presented as mean ± SD and categorical data were presented as number (%)

Table 10 demonstrates that there was a highly statistically significant (*P* value < 0.001) negative strong correlation (*r* = − 0.7) between FFR and MPI. There was a highly statistically significant (*P* value < 0.001) positive strong correlation (*r* = 0.947) between FFR and IFR.

**Table 10:** Correlation between the FFR, MPI, and IFR among the studied patients

FFR value (Cut-off = 0.80)	SPECT-MPI	IFR value (Cut-off = 0.89)
Pearson correlation	− 0.704	0.947
*P* value	< 0.001	< 0.001
*N*	25	25

Table 11 shows that there was a strong (kappa = 0.754) significant (*P* value < 0.001) agreement between the MPI results and FFR results and the overall agreement was 88%. The sensitivity of the MPI was 81.8%, the specificity was 92.9%, the positive predictive value was 90%, the negative predictive value was 86.7%, the positive likelihood ratio was 11.45, and the negative likelihood ratio was 0.20.

**Table 11:** Agreement between the MPI results and the FFR results in the studied patients

SPECT MPI result	FFR result		Total
	+ve	−ve	
+ve	18	2	20
	81.8%	7.1%	40.0%
−ve	4	26	30
	18.2%	92.9%	60.0%
Total	22	28	50
	100.0%	100.0%	100.0%
*P* value	< 0.001**		
Kappa Agreement	0.754		

Data were presented as number and percent

**Fig. 1:**
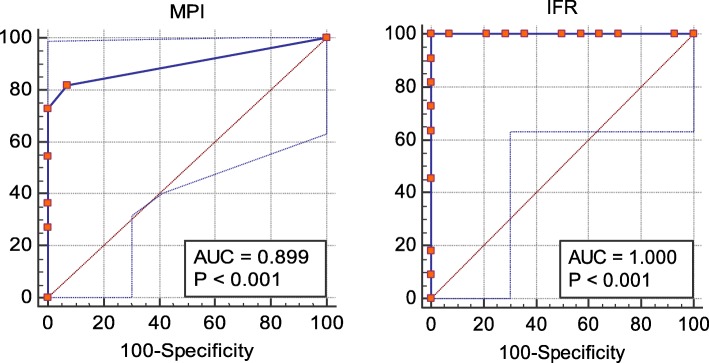
ROC curve for the prediction of positive FFR cases by using the MPI and the IFR

Table 12 shows that there was a strong (kappa = 0.918) significant (*P* value < 0.001) agreement between the IFR results and FFR results and the overall agreement was 96%. The sensitivity of the IFR was 90.9%, the specificity was 100%, the positive predictive value was 100 %, the negative predictive value was 93.3%, and the negative likelihood ratio was 0.09 Table 13 shows ROC curve results for the prediction of positive FFR cases by using the MPI and the IFR and Table 14 shows Summary of results of SPECT - MPI versus IFR side by side Figure 1 shows ROC curve for the prediction of positive FFR cases by using the MPI and the IFR.

**Table 12:** Agreement between the IFR results and the FFR results in the studied patients

IFR result	FFR result		Total
	+ve	−ve	
+ve	20	0	20
	90.9%	0%	40.0%
−ve	2	28	30
	9.1%	100%	60.0%
Total	22	28	50
	100.0%	100.0%	100.0%
*P* value	< 0.001		
Kappa Agreement	0.918		

Data were presented as number and percent

N.B. the positive likelihood ratio was not calculated as the specificity was 100% and the PLR is calculated from the following equation: sensitivity/1−specificity = 90.9/1 − 1 = cannot be calculated

**Table 13:** ROC curve results for the prediction of positive FFR cases by using the MPI and the IFR

Test result variable(s)	Cut-off	Sensitivity	Specificity	The area under the curve	95% confidence interval	
					Lower bound	Upper bound
SPECT_MPI defect size	5	81.8%	92.9%	0.899	0.754	1.000
IFR value cut-off0.89	0.85	90.9%	100%	1.000	0.950	1.000

**Table 14:** Summary of results of SPECT-MPI versus IFR side by side

	SPECT-MPI	IFR
Nature of the test	Non-invasive imaging	Invasive
Pearson correlation to FFR	Strong negative correlation (− 0.704)	Strong positive correlation (0.947)
Kappa agreement to FFR	0.754	0.918
Overall agreement to FFR	88%	96%
Sensitivity	81.8%	90.9%
Specificity	92.9%	100%
Positive predictive value (PPV)	90%	100%
Negative predictive value (NPV)	86.7%	93.3%
Positive likelihood ratio (PLR)	11.45	Cannot be calculated
Negative likelihood ratio (NLR)	0.20	0.09
Area under ROC curve	0.899	1.000
Expected cut-off by ROC curve	Defect size > 5%	> 0.85

## Discussion

Myocardial perfusion imaging by single-photon emission tomography (MPI SPECT) was shown to have high overall sensitivity and specificity estimated at 81.8% and 92.9%, respectively, as well as high positive predictive value (PPV) and negative predictive value (NPV) estimated as 90% and 86.7% respectively. Actually, this concordance between MPI SPECT and FFR results can be already expected and understood from the early validation studies on FFR itself that used MPI SPECT to assess the validity of FFR. In one study; I. Erhard, J. Rieber et al. [14] SPECT MPI and dobutamine stress Echocardiography were used as reference tests to evaluate FFR and by performing ROC analysis, the best cut-off value (highest sum of sensitivity and specificity) was found at 0.75. At this cut-off value using both non-invasive tests as a reference method, sensitivity and specificity were 83% and 77%. Another study, Caymaz O, Fak A et al. [15] prospectively evaluated 40 lesions using a 0.014 in. pressure wire during elective coronary angiography and compared the findings with those of myocardial perfusion 201 Thallium; the study concluded that FFRmyo seems to accurately predict the presence of ischemia on SPECT 201Tl in patients with Stable CAD, while QCA does not reliably assess the physiologic impact of the same lesions. Also, our results are consistent with some small previous studies that used the FFR as the gold standard test. In one study, Morteza Safi et al. [16] published at the Egyptian Heart Journal in 2016, conducted on 45 patients and concluded that there was a significant concordance between FFR and myocardial perfusion imaging for assessment of ischemia, for LAD territory involvement, myocardial perfusion imaging had a sensitivity, specificity, PPV, NPV, and accuracy of nearly 67%, 71%, 31%, 92%, and 70%; for LCX territory involvement, myocardial perfusion imaging had a sensitivity, specificity, PPV, NPV, and accuracy of nearly 100%, 60%, 33%, 100%, and 67% and for RCA territory involvement, myocardial perfusion imaging SPECT had a sensitivity, specificity, PPV, NPV, and accuracy of 100%, 60%, 20%, 100%, and 70%, respectively. In the study by Ilgin Sahiner et al. [17], the Quantitative MPI SPECT analysis compared to FFR has overall sensitivity and specificity of 85% and 84%, respectively, and it was superior to visual analysis.

Tao Zhoua et al. [18] reviewed 13 manuscripts. The pooled data at the vessel level was a sensitivity of 66% (95% CI, 57–74%) and specificity of 81% (95% CI, 70–89%).

In another meta-analysis, Ibrahim Danad et al. [19] evaluated MPI SPECT among other non-invasive tests to determine the diagnostic performance of these tests when compared to the gold standard FFR. At the patient level, 110 patients were involved, the summary sensitivity and specificity were 70% (59–80%) and 78% (68–87%) for MPI SPECT; and the positive and negative likelihood ratios were 3.4 (1.04–11.08) and 0.4 (0.19–0.83). At the vessel-level, 470 vessels were involved, pooled sensitivity was 57% (49–64%) and specificity was 75% (69–80%); the positive and negative likelihood ratios were 2.34 (1.61–3.42) and 0.55 (0.44–0.69). Neng Dai et al. [20] evaluated MPI SPECT among other non-invasive tests to determine the diagnostic performance of these tests when compared to the FFR. The overall sensitivity and specificity were 78% (71–84%) and 79% (70–87%) while the positive and negative likelihood ratios were 3.76 (2.52–5.63) and 0.28 (0.21–0.37). Juhani Knuuti et al. [21] published MPI SPECT overall sensitivity and specificity were 73% (62–82%) and 83% (71–90%) while the positive and negative likelihood ratios were 4.21 (2.62–6.76) and 0.33 (0.24–0.46).

On the other hand, some studies showed poor concordance between MPI SPECT and FFR especially in the patients with multi-vessel disease which was a more challenging situation to MPI SPECT evaluation due to the fact that MPI SPECT actually assesses the relative blood flow differences among vascular territories and so the possibility of balanced ischemia in the presence of more than one vessel disease may be an obstacle for accurate assessment. For example, in Narbeh Melikian et al. study [22] more than 60 patients (nearly 200 vascular territories) with angiographic more than one vessel coronary disease were prospectively scheduled to undergo rest/stress myocardial perfusion imaging and FFR in each vessel; in nearly 42% of patients, MPI and FFR detected identical ischemic territories, in 36%, MPI underestimated and in 22% overestimated the number of ischemic territories in comparison with FFR. There was no an accurate concordance between the ability of the two techniques to detect significant ischemia. On a per-patient basis, there was no accurate concordance between the ability of the two techniques to detect significant ischemia. In comparison with the FFR, the sensitivity, specificity, PPV, and NPV of MPI being able to detect myocardial ischemia was 76%, 38%, 66%, and 50%, respectively. Also on a per-vessel basis, there was no accurate concordance between the MPI and the FFR to detect significant ischemia. Tao Zhou et al. [18] stated that multi-vessel disease leads to the limitation of the myocardial perfusion imaging to assess the functional significance of coronary artery disease in patients with multi-vessel and left main coronary artery disease. In our small study, we excluded multivessel disease to avoid this dilemma especially with the limited resources that served the study on a relatively small sample of patients and we hope that the patients with multi-vessel and left main disease will be targeted in larger studies.

The instantaneous wave-free ratio (IFR) was shown to have sensitivity and specificity estimated as 90.9% and 100%, respectively, as well as positive predictive value (PPV) and negative predictive value (NPV) estimated as 100% and 93.3% respectively. Actually, this concordance between IFR and FFR results can be already expected and understood from the validation studies on IFR which compared both IFR and FFR against different other third-party tests and showed a great concordance between the two methods. In one study: the CLARIFY [23]. In nearly 50 vessels, the IFR, FFR, and HSR (hyperemic stenosis resistance) were compared. The IFR and FFR had an equally good diagnostic agreement with HSR.

In another study; Hwang D, Jeon K-H, et al. [24] included more than 100 consecutive patients with LAD stenosis who underwent both PET scan and invasive physiological assessment then optimal cut-off values of FFR, IFR, and resting Pd/Pa were assessed using PET-derived coronary flow reserve (CFR) and relative flow reserve (RFR) as references. The overall diagnostic accuracy of FFR, IFR, and resting Pd/Pa was not different for CFR < 2.0 (nearly FFR 70%, iFR 74%, and resting Pd/Pa 70%) and RFR < 0.75 (nearly FFR 74%, IFR 71%, and resting Pd/Pa 75%). Also, this concordance continued in large patient outcome trials conducted on both tests especially DEFINE-FLAIR [25] and IFR SWEDEHEART [26].

There are also some small studies were similar to our study design and compared IFR results directly to FFR results as a gold standard reference test.

In one study, Tobias Härle et al. [27] assessed the accuracy of the IFR prospectively in more than 100 patients with borderline coronary lesions; the IFR correlated strongly with the FFR (*r*_s_ = 0.81; *P* < 0.0001). ROC analysis showed an area under the curve (AUC) equals 0.9106, suggesting the high reliability of the IFR as an accurate diagnostic test. The IFR-only technique with a treatment cut-point ≤ 0.89 revealed a diagnostic agreement with the FFR-only technique strategy in more than 120 lesions (nearly 83%) with a sensitivity of nearly 80%, a specificity of 86%. In another study, Alfredo Fede et al. [28] included more than 50 patients with borderline lesions and there was close agreement between the FFR and the IFR (*R* = 0.83, *P* < 0.0001). Deep Chandh Raja et al. [29] found that IFR correlated with FFR in all the subgroups and across all the vessels, without any influence for the heart rate or blood pressure on the correlation with FFR.

Also, there are some meta-analysis studies that addressed this point and clarified this concordance.

In one meta-analysis, Salvatore De Rosa et al. [30] evaluated nearly 6000 lesions. There was a significant correlation between the FFR and IFR of 0.798 (0.78–0.82; *P* < 0.001). Also, they compared IFR and FFR to a third independent reference standard that was invasive coronary flow reserve, non-invasive coronary flow reserve, hyperemic stenosis resistance, a combined reference standard of myocardial perfusion scintigraphy, and hyperemic stenosis resistance index or positron emission tomography perfusion imaging. There were no significant differences between IFR and FFR, both in terms of diagnostic accuracy, measured as the area under the ROC curve and in terms of the diagnostic agreement to the third comparator used in these studies.

In another meta-analysis Rohit Maini et al. [31] nearly 6000 lesions were evaluated. Pooled diagnostic accuracy estimates of IFR versus FFR were: sensitivity 0.78 (95% CI, 0.76–0.79), specificity 0.83 (0.81–0.84), the positive likelihood ratio was 4.54, negative likelihood ratio 0.28 (0.24–0.32), diagnostic odds ratio 17.38 (14.16–21.34), area under the ROC curve was 0.87, and the overall diagnostic accuracy was 0.81.

### Limitations of the study

Despite the encouraging results presented in our small study, there were significant limitations including the small sample size and the exclusion of some types of patients that may be candidates for evaluation as patients with previous myocardial infarction and patients with multi-vessel disease.

## Conclusions

The myocardial perfusion imaging by single-photon emission tomography (MPI SPECT) and instantaneous wave-free ratio (IFR) techniques showed significant concordance with fractional flow reserve (FFR) which is the gold standard reference test for physiological assessment of coronary lesions regardless of the type of diseased coronary artery. In this context, both MPI SPECT and IFR showed high sensitivity, specificity, NPV, and PPV compared with FFR. Therefore, the instantaneous wave-free ratio (IFR) may be a valid alternative to fractional flow reserve to assess the functional significance of intermediate coronary lesions without using agents to induce maximal hyperemia. More importantly, this study may encourage the use of myocardial perfusion imaging by single-photon emission tomography (MPI SPECT) as an alternative, non-invasive, relatively non-expensive test for the evaluation of the physiological significance of intermediate coronary lesions.

We hope and recommend that this study will be a nucleus for further larger studies in the future that may present more solid evidence and target special patient populations excluded from this study as patients with previous myocardial infarction and patients with multi-vessel disease.

## Data Availability

The datasets used and or analyzed during the current study are available from the corresponding author on reasonable request.

## Abbreviations

ACS: Acute coronary syndrome
ADVISE study: Adenosine Vasodilator Independent Stenosis Evaluation study
ATP: Adenosine tri-phosphate
AUC: Area under the curve
BMI: Body mass index
CA: Coronary angiography
CABG: Coronary artery bypass grafting
CAD: Coronary artery disease
CFR: Coronary flow reserve
CI: Confidence interval
CLARIFY study: Classification Accuracy of Pressure Only Ratios Against Indices Using Flow study
CMR: Cardiac magnetic resonance
COMPAREACUTE study: Comparison Between FFR Guided Revascularization Versus Conventional Strategy in Acute STEMI Patients With MVD
DEFER study: Deferral versus performance of PTCA [percutaneous transluminal coronary angioplasty] in patients without documented ischemia
DEFINE-FLAIR study: Functional Lesion Assessment of Intermediate Stenosis to Guide Revascularization
DES: Drug-eluting stent
DM: Diabetes mellitus
DSE: Dobutamine stress echocardiogram
DSVE: Diameter stenosis by visual estimation
DSQCA: Diameter stenosis by quantitative coronary angiography
ESC: European Society of Cardiology
FAME study: Fractional Flow Reserve versus Angiography for Multivessel Evaluation
FFR: Fractional flow reserve
FFRcor: Coronary fractional flow reserve
FFRmyo: Myocardial fractional flow reserve
HbA1C: Hemoglobin A1C=glycated hemoglobin
HR: Hazard ratio
HSR: Hyperemic stenosis resistance
HTN: Hypertension
IDEAL study: Iberian-Dutch–English study
IFR: Instantaneous wave-free ratio
IFR SWEDEHEART study: Evaluation of IFR vs FFR in Stable Angina or Acute Coronary Syndrome
ISR: In-stent restenosis
LAD: Left anterior descending (artery)
LCX: Left circumflex (artery)
LMCA: Left main coronary artery
LVH: Left ventricular hypertrophy
MACE: Major adverse cardiac event
MI: Myocardial infarction
MPI: Myocardial perfusion imaging
MPS: Myocardial perfusion scan
MVD: Multi-vessel disease
NLR: Negative likelihood ratio
NPV: Negative predictive value
OGTT: Oral glucose tolerance test
OMT: Optimal medical therapy
Pa: Pressure proximal to stenosis or pressure in aorta
PCI: Percutaneous coronary intervention
Pd: Pressure distal to a stenosis
PET: Positron emission tomography
PLR: Positive likelihood ratio
PPV: Positive predictive value
PTCA: Percutaneous transluminal coronary angioplasty
Pv: Right atrial pressure
Pw: Coronary wedge pressure
RCA: Right coronary artery
RR: Relative risk
SCAR: Swedish Coronary Angiography and Angioplasty Registry
SD: Standard deviation
SPECT: Single-photon emission tomography
STEMI: ST-segment elevation myocardial infarction
SVD: Single-vessel disease
WIA: Wave intensity analysis
WFP: Wave-free period
X-ECG: Exercise electrocardiogram

## References

[1] Berry C, Corcoran D, Hennigan B, Watkins S, Layland J, Oldroyd KG (2015). Fractional flow reserve-guided management in stable coronary disease and acute myocardial infarction: recent developments. European heart journal..

[2] Meier B, Gruentzig AR, Goebel N, Pyle R, von Gosslar W, Schlumpf M (1983). Assessment of stenoses in coronary angioplasty. Inter-and intraobserver variability. Int J Cardiol..

[3] Detre KM, Wright E, Murphy ML, Takaro T (1975). Observer agreement in evaluating coronary angiograms. Circulation..

[4] Zir LM, Miller SW, Dinsmore RE, Gilbert JP, Harthorne JW (1976). Interobserver variability in coronary angiography. Circulation..

[5] Iguchi T, Hasegawa T, Nishimura S, Nakata S, Kataoka T, Ehara S (2013). Impact of lesion length on functional significance in intermediate coronary lesions. Clinical cardiology..

[6] Götberg M, Cook CM, Sen S, Nijjer S, Escaned J, Davies JE (2017). The evolving future of instantaneous wave-free ratio and fractional flow reserve. Journal of the American College of Cardiology..

[7] Henzlova MJ, Duvall WL, Einstein AJ, Travin MI, Verberne HJ (2016). ASNC imaging guidelines for SPECT nuclear cardiology procedures: Stress, protocols, and tracers. Journal of Nuclear Cardiology..

[8] Association WM (2013). World Medical Association Declaration of Helsinki: ethical principles for medical research involving human subjects. JAMA..

[9] Lang RM, Badano LP, Mor-Avi V, Afilalo J, Armstrong A, Ernande L (2015). Recommendations for cardiac chamber quantification by echocardiography in adults: an update from the American Society of Echocardiography and the European Association of Cardiovascular Imaging. Eur Heart J Cardiovasc Imaging..

[10] Tilkemeier PL, Bourque J, Doukky R, Sanghani R, Weinberg RL (2017). ASNC imaging guidelines for nuclear cardiology procedures. J Nucl Cardiol..

[11] Toth GG, Johnson NP, Jeremias A, Pellicano M, Vranckx P, Fearon WF (2016). Standardization of fractional flow reserve measurements. J Am Coll Cardiol..

[12] Matsumoto H, Nakatsuma K, Shimada T, Ushimaru S, Mikuri M, Yamazaki T (2014). Effect of caffeine on intravenous adenosine-induced hyperemia in fractional flow reserve measurement. The Journal of invasive cardiology..

[13] Adjedj J, Toth GG, Johnson NP, Pellicano M, Ferrara A, Floré V (2015). Intracoronary adenosine: dose–response relationship with hyperemia. JACC Cardiovasc Interv..

[14] Erhard I, Rieber J, Jung P, Hacker M, Schiele T, Stempfle H-U (2005). The validation of fractional flow reserve in patients with coronary multivessel disease: a comparison with SPECT and contrast-enhanced dobutamine stress echocardiography. Zeitschrift für Kardiologie..

[15] Caymaz O, Fak A, Tezcan H, Inanir S, Toprak A, Tokay S (2000). Correlation of myocardial fractional flow reserve with thallium-201 SPECT imaging in intermediate-severity coronary artery lesions. J Invasive Cardiol..

[16] Safi M, Karimlu MR, Khaheshi I, Ataeinia B (2016). Concordance between myocardial perfusion scan assessed by SPECT and fractional flow reserve findings for detection of significant ischemia. Egypt Heart J..

[17] Sahiner I, Akdemir UO, Kocaman SA, Sahinarslan A, Timurkaynak T, Unlu M (2013). Quantitative evaluation improves specificity of myocardial perfusion SPECT in the assessment of functionally significant intermediate coronary artery stenoses: a comparative study with fractional flow reserve measurements. Annals of nuclear medicine..

[18] Zhou T, Yang L-F, Zhai J-L, Li J, Wang Q-M, Zhang R-J (2014). SPECT myocardial perfusion versus fractional flow reserve for evaluation of functional ischemia: a meta analysis. Eur J Radiol..

[19] Danad I, Szymonifka J, Twisk JW, Norgaard BL, Zarins CK, Knaapen P (2017). Diagnostic performance of cardiac imaging methods to diagnose ischaemia-causing coronary artery disease when directly compared with fractional flow reserve as a reference standard: a meta-analysis. European heart journal..

[20] Dai N, Zhang X, Zhang Y, Hou L, Li W, Fan B (2016). Enhanced diagnostic utility achieved by myocardial blood analysis: a meta-analysis of noninvasive cardiac imaging in the detection of functional coronary artery disease. International journal of cardiology..

[21] Knuuti J, Ballo H, Juarez-Orozco LE, Saraste A, Kolh P, Rutjes AWS (2018). The performance of non-invasive tests to rule-in and rule-out significant coronary artery stenosis in patients with stable angina: a meta-analysis focused on post-test disease probability. European heart journal..

[22] Melikian N, De Bondt P, Tonino P, De Winter O, Wyffels E, Bartunek J (2010). Fractional flow reserve and myocardial perfusion imaging in patients with angiographic multivessel coronary artery disease. JACC Cardiovasc Interv..

[23] Sen S, Asrress KN, Nijjer S, Petraco R, Malik IS, Foale RA (2013). Diagnostic classification of the instantaneous wave-free ratio is equivalent to fractional flow reserve and is not improved with adenosine administration: results of CLARIFY (Classification Accuracy of Pressure-Only Ratios Against Indices Using Flow Study). J Am Coll Cardiol..

[24] Hwang D, Jeon K-H, Lee JM, Park J, Kim CH, Tong Y (2017). Diagnostic performance of resting and hyperemic invasive physiological indices to define myocardial ischemia: validation with 13N-ammonia positron emission tomography. JACC Cardiovasc Interv..

[25] Davies JE, Sen S, Dehbi H-M, Al-Lamee R, Petraco R, Nijjer SS (2017). Use of the instantaneous wave-free ratio or fractional flow reserve in PCI. N Engl J Med..

[26] Götberg M, Christiansen EH, Gudmundsdottir IJ, Sandhall L, Danielewicz M, Jakobsen L (2017). Instantaneous wave-free ratio versus fractional flow reserve to guide PCI. N Engl J Med..

[27] Härle T, Bojara W, Meyer S, Elsässer A (2015). Comparison of instantaneous wave-free ratio (iFR) and fractional flow reserve (FFR)—first real world experience. International journal of cardiology..

[28] Fede A, Zivelonghi C, Benfari G, Pesarini G, Pighi M, Ferrara A (2015). iFR-FFR comparison in daily practice: a single-center, prospective, online assessment. J Cardiovasc Med..

[29] Raja DC, Subban V, Mathew R, Abdullakutty J, Joseph J, George J (2019). Comparison of resting and adenosine-free pressure indices with adenosine-induced hyperemic fractional flow reserve in intermediate coronary lesions. Indian heart journal..

[30] De Rosa S, Polimeni A, Petraco R, Davies JE, Indolfi C (2018). Diagnostic performance of the instantaneous wave-free ratio: comparison with fractional flow reserve. Circulation: Cardiovascular Interventions..

[31] Maini R, Moscona J, Katigbak P, Fernandez C, Sidhu G, Saleh Q (2018). Instantaneous wave-free ratio as an alternative to fractional flow reserve in assessment of moderate coronary stenoses: A meta-analysis of diagnostic accuracy studies. Cardiovasc Revasc Med..

